# Analysis of the possible cytogenetic mechanism for overcoming hybrid lethality in an interspecific cross between *Nicotiana suaveolens* and *Nicotiana tabacum*

**DOI:** 10.1038/s41598-021-87242-7

**Published:** 2021-04-09

**Authors:** Kouki Nakata, Hiroki Nagashima, Natsuki Inaba, Haruka Yamashita, Yoshihito Shinozaki, Motoki Kanekatsu, Wataru Marubashi, Tetsuya Yamada

**Affiliations:** 1 Graduate School of Agricultural Science, Tokyo University of Agriculture and Technology, Tokyo, 183-0054 Japan; 2 Faculty of Agricultural Science, Meiji University, Kanagawa, Japan; 3 Present Address: Division of Evolutionary Genetics, National Institute of Genetics, Shizuoka, Japan; 4 Present Address: Department of Genetics, The Graduate University for Advanced Studies (SOKENDAI), Shizuoka, Japan

**Keywords:** Genomic instability, Plant breeding, Plant reproduction

## Abstract

Hybrid lethality is a type of reproductive isolation in which hybrids die before maturation, due to the interaction between the two causative genes derived from each of the hybrid parents. The interspecific hybrid of *Nicotiana suaveolens* × *Nicotiana tabacum* is a model plant used in studies on hybrid lethality. While most of the progeny produced from such a cross die, some individuals grow normally and mature. Separately, a technique for producing mature hybrids by artificial culture has been developed. However, the mechanism by which hybrids overcome lethality, either spontaneously or by artificial culture, remains unclear. In the present study, we found that some hybrids that overcome lethality, either spontaneously or by artificial culture, lack the distal part of the Q chromosome, a region that includes the gene responsible for lethality. Quantitative polymerase chain reaction results suggested that the distal deletion of the Q chromosome, detected in some hybrid seedlings that overcome lethality, is caused by reciprocal translocations between homoeologous chromosomes. The results showed that chromosomal instability during meiosis in amphidiploid *N*. *tabacum* as well as during artificial culturing of hybrid seedlings is involved in overcoming hybrid lethality in interspecific crosses of the genus *Nicotiana*.

## Introduction

Hybrid lethality is a type of postzygotic reproductive isolation in which fertilization between different species or populations occurs, but the resulting hybrids do not mature. In higher plants that experience hybrid lethality, fertilization is successful, but the hybrid embryos either die before germination or the hybrid seedlings exhibit lethal symptoms, such as browning, withering, and yellowing after germination. Hybrid lethality has been reported in a wide range of plant species, including wheat^1^, rice^2^, and cotton^3^. This phenomenon is a significant obstacle that restricts the genetic resources available for use in plant cross-breeding.

Hybrid lethality has been demonstrated in various combinations of interspecific crosses in the genus *Nicotiana*^4^. Hybrid seedlings of *Nicotiana suaveolens* (2n = 2 × = 32, genome constitution SuSu) and *N. tabacum* (2n = 4 × = 48, SSTT, the S genome is derived from *N. sylvestris*, and the T genome is derived from *N. tomentosiformis*^5^) germinate normally, but show type-II lethality, with the hypocotyl turning brown and dying several days after germination (DAG)^6^. It has been reported that the amount of transcript of *PAL*, an immune response-related gene that encodes phenylalanine ammonia-lyase, is increased in hybrid seedlings that show lethality^6^. We also have confirmed that the accumulation of protein aggregates promotes programmed cell death via autophagy in cultured cells of *N. suaveolens* × *N. tabacum* hybrids that exhibit lethality^7,8^.

Hybrid lethality is caused by the interaction of two complementary genes derived from each hybrid parent^9,10^. In plants, *R* genes encoding proteins that recognize effectors derived from a pathogen during a disease response have been reported to cause hybrid lethality^11–13^. In the genus *Nicotiana*, *Nt6549g30*, a kind of NBS-LRR-type *R* gene of *N*. *tabacum*, is responsible for the type-II lethality expressed in hybrid seedlings from crosses between *N*. *tabacum* and any of nine wild species, except *N*. *suaveolens*, in *Nicotiana* section *Suaveolentes*^12,14^. On the other hand, the locus responsible for lethality in the *N*. *suaveolens* × *N*. *tabacum* cross is located on the Q chromosome in the S genome of *N*. *tabacum*^15–17^.

Previous studies have reported conflicting findings regarding the chromosomal location of *Nt6549g30*. Ma^12^ reported that *Nt6549g30* is present on the H chromosome, while another study suggested that the H chromosome belongs to the T genome^18^. On the other hand, a single sequence repeat (SSR) marker for Linkage group No. 11, a marker that is detected on the Q chromosome of the S genome^19^, was also detected on the H chromosome^20^. Additionally, Ma^12^ reported that the H chromosome belongs to the S genome. Consequently, we consider that the H chromosome in the reports of Hancock et al*.*^20^ and Ma^11^ is the same as the Q chromosome in Tezuka et al*.*^19^. If this interpretation is valid, then we consider it highly likely that *Nt6549g30* is involved in the lethality of the *N. suaveolens* × *N. tabacum* cross.

In some lethal cross combinations between *N. tabacum* and wild *Nicotiana* species, including *N*. *suaveolens* × *N*. *tabacum*, viable hybrid seedlings (i.e., hybrid seedlings that overcome lethality) appear spontaneously at a certain frequency. Hancock et al*.*^20^ detected hybrid seedlings that overcame lethality at a frequency of approximately 1.1 × 10^−3^ in a *N*. *tabacum* × *N*. *africana* cross. They also confirmed that the SSR marker at the distal part of Linkage group No. 11 was not detected in approximately 47% of such seedlings, suggesting the loss of the distal segment of the H chromosome (Q chromosome). This missing chromosomal end region contains *Nt6549g30* (reference 11). However, the cause of such high-frequency loss of a chromosome terminus in hybrid seedlings remains unknown.

It has been reported that reciprocal translocation can occur between homoeologous chromosomes during meiosis in allopolyploid plant species such as *Brassica napus*^21^, coffee^22^, and interspecific hybrids of the genus *Lilium*^23^. In *N*. *tabacum*, reciprocal translocation has also been reported to occur between homoeologous chromosomes, resulting in loss of the *N* gene, a kind of *R* gene^24^. Based on these observations, we hypothesized that hybrid seedlings that overcome lethality following a cross between *N. tabacum* and wild species are produced by the formation of mutated gametes in which a distal segment of the Q chromosome, including *Nt6549g30*, is replaced with the homologous region of the homoeologous Q′ chromosome by reciprocal translocation during meiosis (Hypothesis 1).

Since hybrid lethality is an obstacle to cross-breeding, various methods have been employed to produce hybrid plants that overcome lethality. In intra- and interspecific hybrids of wheat, hybrid plants that overcome lethality have been obtained by artificial culture of hybrid embryos^25^, by proline treatment during fertilization, and hybrid embryogenesis^26^. In *N. suaveolens* × *N. tabacum*, when hybrid seedlings were cultured in a medium containing a high concentration of cytokinin, vigorous shoots were formed at the stem bases of the seedlings, and regenerated plants that overcame lethality were obtained by rooting of these shoots^27^. However, the mechanism whereby plants that overcome lethality are produced by artificial culturing remains unclear.

In in vitro cultured explants, it is known that reactive oxygen species (ROS) are generated by oxidative stress induced by medium components (plant hormones and salts) and the culture environment^28^. These ROS are also considered to cause chromosome breakage^29^. Based on the above observations, we hypothesized that the production of regenerated *N*. *suaveolens* × *N*. *tabacum* hybrid seedlings that overcome lethality after culturing in cytokinin-supplemented medium results from the appearance of cells lacking the distal part of the Q chromosome during in vitro culture (Hypothesis 2).

Elucidating the causes of interspecific hybrids that overcome lethality is expected to reveal one aspect of the mechanism by which new species form by overcoming reproductive isolation. This information also is expected to contribute to the establishment of techniques for overcoming hybrid lethality, thereby leading to an expansion of the genetic resources available for cross-breeding. In the present study, we tested the above two hypotheses in an attempt to clarify the mechanisms by which hybrid plants overcome lethality. First, we used the polymerase chain reaction (PCR) to identify the presence or absence of *Nt6549g30* and the distal part of the Q chromosome in seedlings and regenerated plants derived from a *N*. *suaveolens* × *N*. *tabacum* cross, that overcame hybrid lethality. Next, we used quantitative PCR (qPCR) to confirm reciprocal translocation between the Q chromosome region where *Nt6549g30* is located and the homologous region of the homoeologous Q′ chromosome.

## Results

### Acquisition of hybrid seedlings that overcome lethality

**Figure 1:**
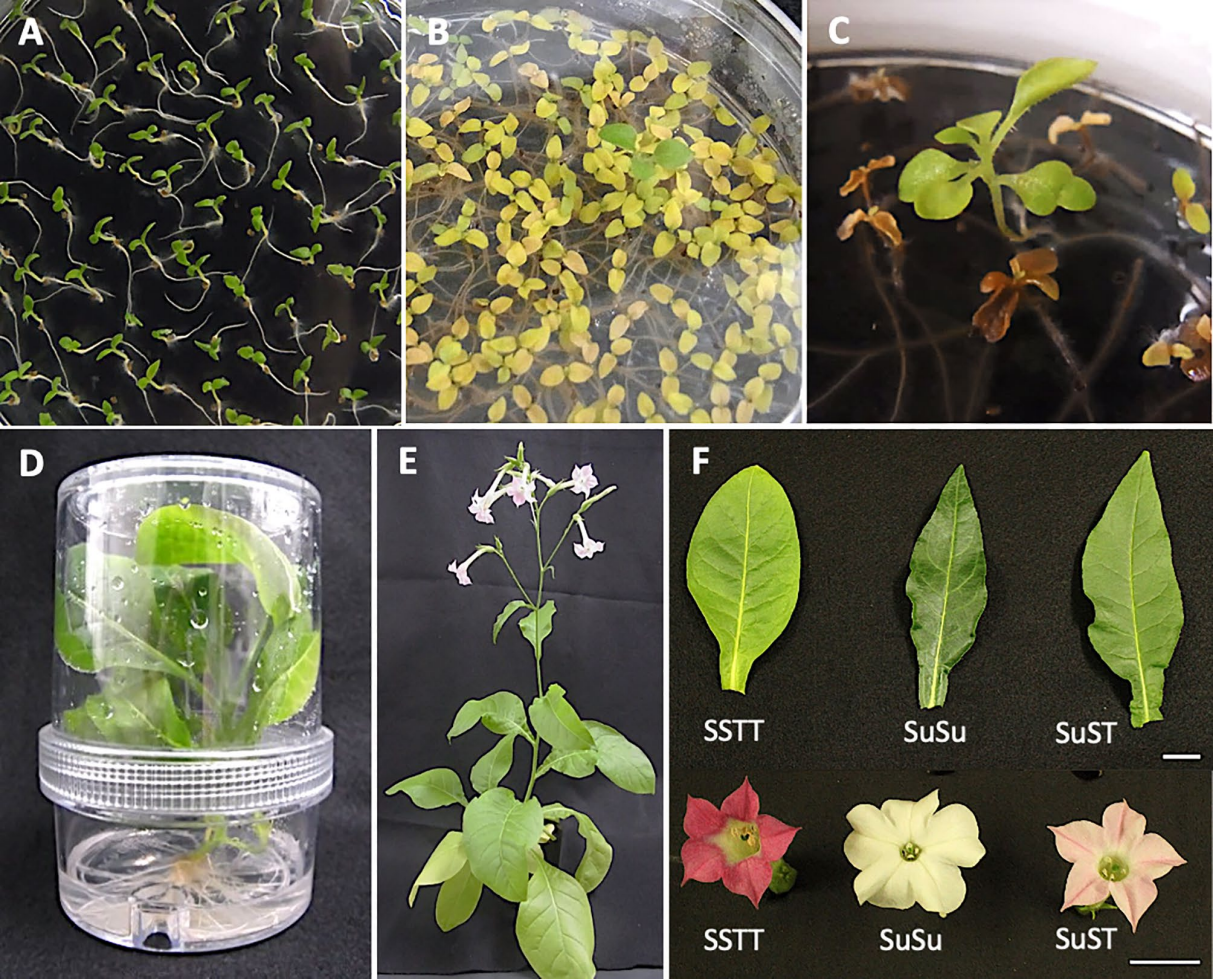
Process of obtaining hybrid viable seedlings. (**A**) Hybrid seedlings at 3 days after germination (DAG). (**B**) Seedlings at 14 DAG. (**C**) Seedling not showing lethality at 20 DAG. (**D**) Viable hybrid seedling cultured and propagated in a plant box. (E) Matured viable plant. (F) Leaf and flower phenotype of parents and hybrid plant. SSTT: *N. tabacum*, SuSu: *N. suaveolens*, SuST: *N. suaveolens* × *N. tabacum*. Scale bar: 2 cm.

**Figure 2:**
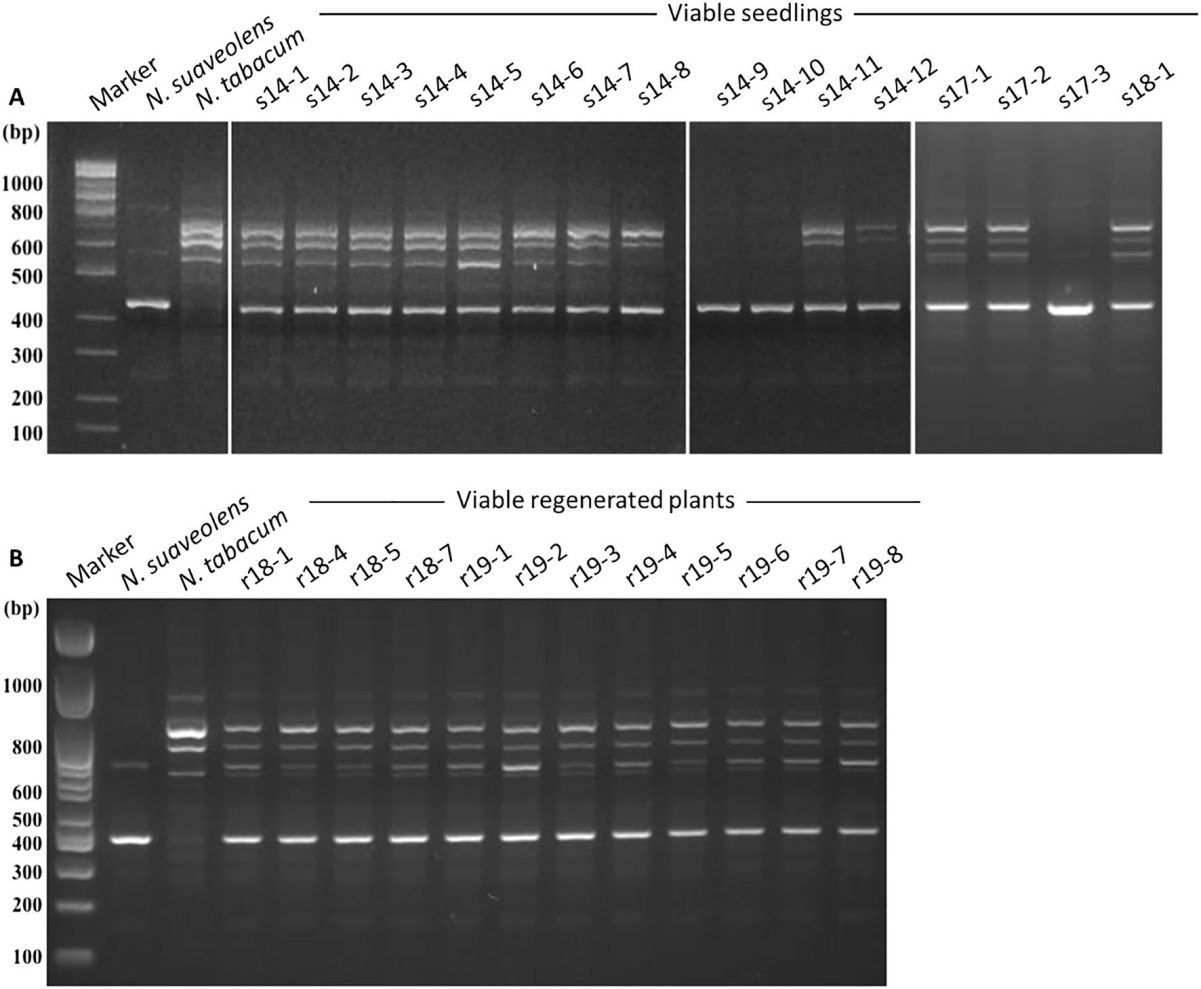
Confirmation of hybridity of viable seedlings and viable regenerated plants of the *N. suaveolens* × *N. tabacum* cross. Products of randomly amplified polymorphic DNA-polymerase chain reaction (RAPD-PCR) performed with primer OPA-12 were separated by agarose gel electrophoresis to assess hybridity of viable seedlings (**A**) and viable regenerated plants (**B**). See also Table S1.

Of 15,476 seeds that were obtained by crossing *N. suaveolens* × *N. tabacum*, 12,943 seeds germinated. Shortly after germination, most of the seedlings from this cross showed lethal symptoms, such as browning of hypocotyls and roots along with yellowing of leaves, but 16 seedlings did not show any lethal symptoms at 20 days after germination (Fig. 1A–C). These seedlings were transferred to half-strength Murashige and Skoog medium (0.5 × MS) in a plant box (Fig. 1D). The Randomly Amplified Polymorphic DNA-PCR (RAPD-PCR) method was used to evaluate the hybridity of viable seedlings; gel electrophoresis of the products showed that 12 of the 16 surviving seedlings yielded all amplicons from both parents (i.e., RAPD bands specific to *N. suaveolens* as well as those specific to *N. tabacum*) (Fig. 2, Table S1). Among the 4 remaining viable seedlings, one seedling (s14-5) lacked one of the three *N*. *tabacum*-specific RAPD bands; this band corresponded to the product obtained with the OPA-15 primer. The other 3 seedlings (s14-9, s14-10, s17-3) yielded only the *N*. *suaveolens*-specific RAPD bands.

**Figure 3:**
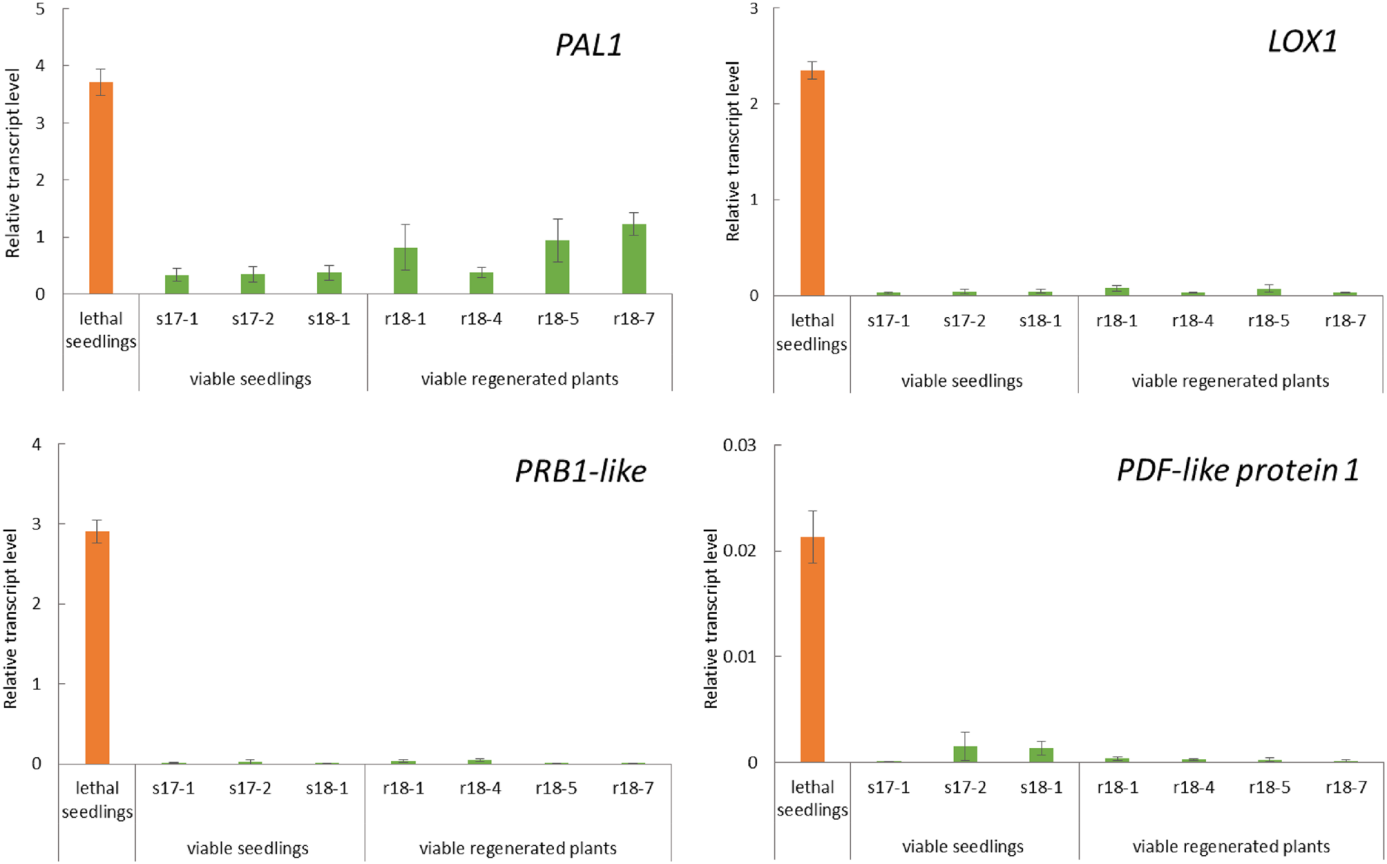
Relative transcript levels of immune response-related genes in cotyledons of lethal seedlings (at 6 days after germination (DAG)) and in young true leaves of viable seedlings (s17-1, s17-2, and s18-1) and viable regenerated plants (r18-1, r18-4, r18-5, and r18-7) of the *N. suaveolens* x × *N. tabacum* cross. Quantitative Real-Time-Polymerase Chain Reaction (qRT-PCR) was used to determine transcript levels of *PAL* (*Phenylalanine ammonia lyase*) *1*, *PRB* (*Basic form of Pathogenesis-related protein*) *-1 like*, *LOX* (*Lipoxygenase*) *1*, *PDF* (*Plant defensin*) *-like protein 1* relative to those of *RFC3* (a housekeeping gene). Data are shown as the mean ± SE from triplicate samples (n = 3) for each treatment.

Fifteen surviving seedlings (with the exception of s17-3) were potted, of which 13 underwent anthesis (Fig. 1E). Eleven of the flowering seedlings were judged to be hybrid seedlings that overcame lethality, given that these seedlings showed morphologies that were intermediate between those of the parental species in terms of flower color and shape, leaf size and shape, and plant posture (Fig. 1F, Table S2). The other two seedlings (s14-9 and s14-10) showed *N. suaveolens*-type flower color and plant posture, which was consistent with the results of the RAPD analysis. For 3 of the 15 surviving seedlings (s17-1, s17-2, and s18-1), transcripts of immune response-related genes (Table S3) were present (in the young true leaves) at levels much lower than those detected in the cotyledons of lethal seedlings at 6 DAG (Fig. 3). Based on these results, the 11 seedlings that showed morphologies intermediate between their parents were judged to be hybrids that had overcome lethality. By this assessment, the frequency of appearance of hybrid seedlings that overcame lethality was 8.5 × 10^−4^.

### Production of regenerated hybrid plants that overcome lethality by in vitro culturing of hybrid seedlings

**Figure 4:**
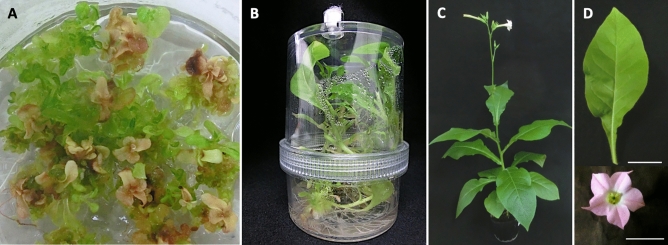
Process of obtaining hybrid regenerated plants not showing lethality. (**A**) Viable shoots formed on hybrid seedlings cultured on medium supplemented with 2.0 mg/L 6-benzylaminopurine (BAP). (**B**) Regenerated plants from viable shoots propagated in a plant box. (**C**) Matured regenerated plant. (**D**) Leaf and flower phenotype of one regenerated plant. Scale bar: 2 cm.

All 35 seedlings produced from the *N*. *suaveolens* × *N*. *tabacum* cross that were cultured in 0.5 × MS medium containing 6-benzylaminopurine (BAP), a synthetic cytokinin, showed lethal symptoms shortly after germination. However, approximately 1 month later, many green shoots were observed to have formed at the bases of the stems of the seedlings (Fig. 4A). Shoots were cut from multiple plants; of 18 randomly selected shoots that were transplanted into 0.5 × MS medium, 14 were able to root (Fig. 4B); those that did not take root vitrified and eventually died. When the hybridity of 12 regenerated plants that grew normally without vitrification were evaluated by the RAPD method, 10 individuals yielded all amplicons from the two parents (i.e., RAPD bands specific to *N. suaveolens* as well as those specific to *N. tabacum*) (Table S1, Fig. 2). In the remaining two regenerated plants (r18-1 and r19-4), one of the two *N*. *tabacum*-specific RAPD bands (i.e., one that was obtained using the OPA-11 primer) was not observed (Table S1).

There was no difference in morphology among the 12 regenerated plants; of 4 of these plants (r18-1, r18-4, r18-5, and r18-7) that were potted, all underwent anthesis (Fig. 4C). All of the regenerated progeny that produced flowers showed morphologies that were intermediate between those of the two parents in terms of flower color and shape, leaf size and shape, and plant posture (Fig. 4D, Table S2). As in the primary seedlings that overcame lethality, the abundance of transcripts of immune response-related genes in young true leaves of the regenerated flowering plants was notably lower than that in the cotyledons of seedlings that died at 6 DAG (Fig. 3). Based on these results, these regenerated plants were judged to be hybrids that that had overcome lethality.

### PCR confirmation of deletion of the distal part of the Q chromosome and *Nt6549g30*

**Figure 5:**
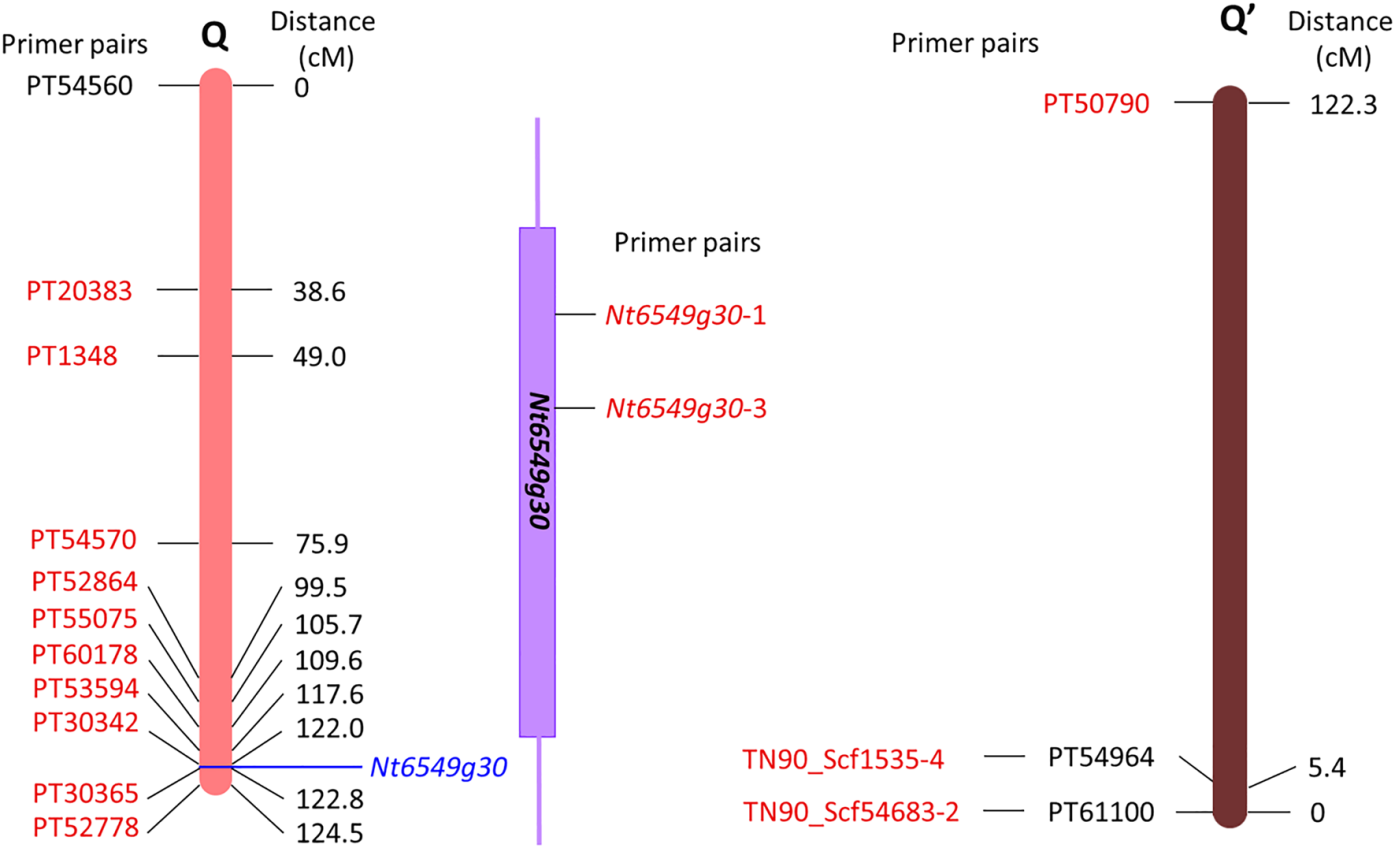
Locations of primer pairs on the Q chromosome (Linkage group No. 11), *Nt6549g30*, and the Q′ (Q homologous) chromosome (Linkage group No. 13). The primer pairs used in this study are indicated in red, whereas others are indicated in black. *Nt6549g30* is closely linked to PT30342, as confirmed by linkage analysis^12^. Distance (cM) indicates genetic distance from the primer pair PT54560 on the Q chromosome, and from the primer pair PT61100 on the Q′ chromosome. TN90_Scf1535-4 and TN90_Scf54683-2 are located close to PT54964 and PT61100, respectively (within ± 31 kbp). See also Table S5 for primer list.

PCR was performed using primers that were specific for the SSR and *Nt6549g30* markers present on the Q chromosome of the S genome of *N*. *tabacum* (Fig. 5). In lethal hybrid seedlings were detected with all SSR primer pairs and *Nt6549g30* primer pairs (Table 1). On the other hand, in the 5 of 8 individual hybrid seedlings that overcame lethality and in 3 of 12 individual viable regenerated hybrid plants that overcame lethality, no amplicon was detected for reactions using the primer pairs (PT30342, PT30365, and PT52778) targeting three SSRs in the distal part of the Q chromosome. Similarly, no amplicons were detected in these 8 individual hybrid seedlings when using the two primer pairs that amplify *Nt6549g30*. In addition, in 6 of the 8 individual hybrid seedlings, several other SSRs were not amplified, but the location and number of the missing SSRs differed among individuals.

**Table 1:** Detection of products, amplified by SSR and *Nt6549g30* primer pairs, from the Q chromosome (Linkage group No. 11) of *N. tabacum* in lethal seedlings, viable seedlings, and viable regenerated plants of the *N. suaveolens* × *N. tabacum* cross.

Type of primer pair	Name of primer pair	Distance(cM)^c^	*N. suaveolens*	*N. tabacum*	Lethal seedlings			Viable seedlings							
					1	2	3	s14-1	s14-2	s14-4	s14-7	s14-8	s17-1	s17-2	s18-1
SSR^a^	PT20383	38.6	**−**	**+**	**+**	**+**	**+**	**+**	**+**	**+**	**+**	**+**	**+**	**+**	**+**
	PT1348	49.0	**−**	**+**	**+**	**+**	**+**	**+**	**+**	**+**	**+**	**+**	**+**	**+**	**+**
	PT54570	75.9	**−**	**+**	**+**	**+**	**+**	**+**	**+**	**+**	**+**	**+**	**+**	**+**	**+**
	PT52864	99.5	**−**	**+**	**+**	**+**	**+**	**+**	**+**	**+**	**+**	**+**	**−**	**+**	**+**
	PT55075	105.7	**−**	**+**	**+**	**+**	**+**	**+**	**+**	**+**	**+**	**+**	**−**	**+**	**+**
	PT60178	109.6	**−**	**+**	**+**	**+**	**+**	**+**	**+**	**+**	**+**	**+**	**−**	**+**	**+**
	PT53094	117.6	**−**	**+**	**+**	**+**	**+**	**+**	**+**	**+**	**+**	**−**	**−**	**−**	**−**
	PT30342	122.0	**−**	**+**	**+**	**+**	**+**	**+**	**+**	**+**	**−**	**−**	**−**	**−**	**−**
	PT30365	122.8	**−**	**+**	**+**	**+**	**+**	**+**	**+**	**+**	**−**	**−**	**−**	**−**	**−**
	PT52778	124.5	**−**	**+**	**+**	**+**	**+**	**+**	**+**	**+**	**−**	**−**	**−**	**−**	**−**
Gene	*Nt6549g30*-1^b^	N.A	**−**	**+**	**+**	**+**	**+**	**+**	**+**	**+**	**−**	**−**	**−**	**−**	**−**
	*Nt6549g30*-3^b^		**−**	**+**	**+**	**+**	**+**	**+**	**+**	**+**	**−**	**−**	**−**	**−**	**−**

Type of primer pair	Name of primer pair	Distance(cM)^c^	Viable regenerated plants												
			r18-1	r18-4	r18-5	r18-7	r19-1	r19-2	r19-3	r19-4	r19-5	r19-6	r19-7	r19-8	
SSR^a^	PT20383	38.6	**+**	**+**	**+**	**+**	**+**	**+**	**+**	**+**	**+**	**+**	**+**	**+**	
	PT1348	49.0	**+**	**+**	**+**	**-**	**+**	**+**	**+**	**+**	**+**	**+**	**+**	**+**	
	PT54570	75.9	**+**	**+**	**+**	**+**	**+**	**+**	**+**	**+**	**+**	**+**	**+**	**+**	
	PT52864	99.5	**−**	**+**	**+**	**-**	**+**	**+**	**+**	**+**	**+**	**+**	**+**	**+**	
	PT55075	105.7	**−**	**+**	**+**	**-**	**+**	**+**	**+**	**+**	**+**	**+**	**+**	**+**	
	PT60178	109.6	**−**	**+**	**+**	**-**	**+**	**+**	**+**	**+**	**+**	**+**	**+**	**+**	
	PT53094	117.6	**−**	**+**	**+**	**-**	**+**	**+**	**+**	**+**	**+**	**+**	**+**	**+**	
	PT30342	122.0	**−**	**+**	**+**	**-**	**+**	**+**	**-**	**+**	**+**	**+**	**+**	**+**	
	PT30365	122.8	**−**	**+**	**+**	**-**	**+**	**+**	**-**	**+**	**+**	**+**	+	**+**	
	PT52778	124.5	**−**	**+**	**+**	**-**	**+**	**+**	**-**	**+**	**+**	**+**	**+**	**+**	
Gene	*Nt6549g30*-1^b^	N.A	**−**	**+**	**+**	**-**	**+**	**+**	**-**	**+**	**+**	**+**	**+**	**+**	
	*Nt6549g30*-3^b^		**−**	**+**	**+**	**-**	**+**	**+**	**-**	**+**	**+**	**+**	**+**	**+**	

‘ + ’ indicates the presence of allele of *N. tabacum*, ‘-’ indicates the absence of allele of *N. tabacum*. N.A., not applicable.

^a^Bindler et al. ^43,44^.

^b^Closely linked to PT30342, as confirmed by linkage analysis (Ma^12^).

^c^Genetic distance from the marker amplified by primer pair PT54560.

Genome analysis was performed using Genotyping by Random Amplicon Sequencing-Direct (GRAS-Di)^30^ on the seedlings of hybrid parents (*N. suaveolens* and *N. tabacum*), two lethal hybrid seedlings (samples No. 1 and 3), and one hybrid seedling that overcame lethality (s17-2). In the GRAS-Di analysis of the amplicons obtained with 63 random primers and mapping to reference sequences, 187 amplicons derived from Q-chromosome DNA in *N*. *tabacum* and lethal hybrid seedlings were detected (Table S4). On the other hand, in the hybrid seedling that overcame lethality (s17-2), amplicons derived from DNA sequences located from bp 346,510 to 81,360,673 on the Q chromosome were detected. However, four amplicons (AMP0045831, AMP0067003, AMP0062028, and AMP0042233) derived from DNA sequences located between bp 81,360,673 and 81,497,158 on the Q chromosome were not detected.

### Confirmation of reciprocal translocation between homoeologous chromosomes by qPCR

**Figure 6:**
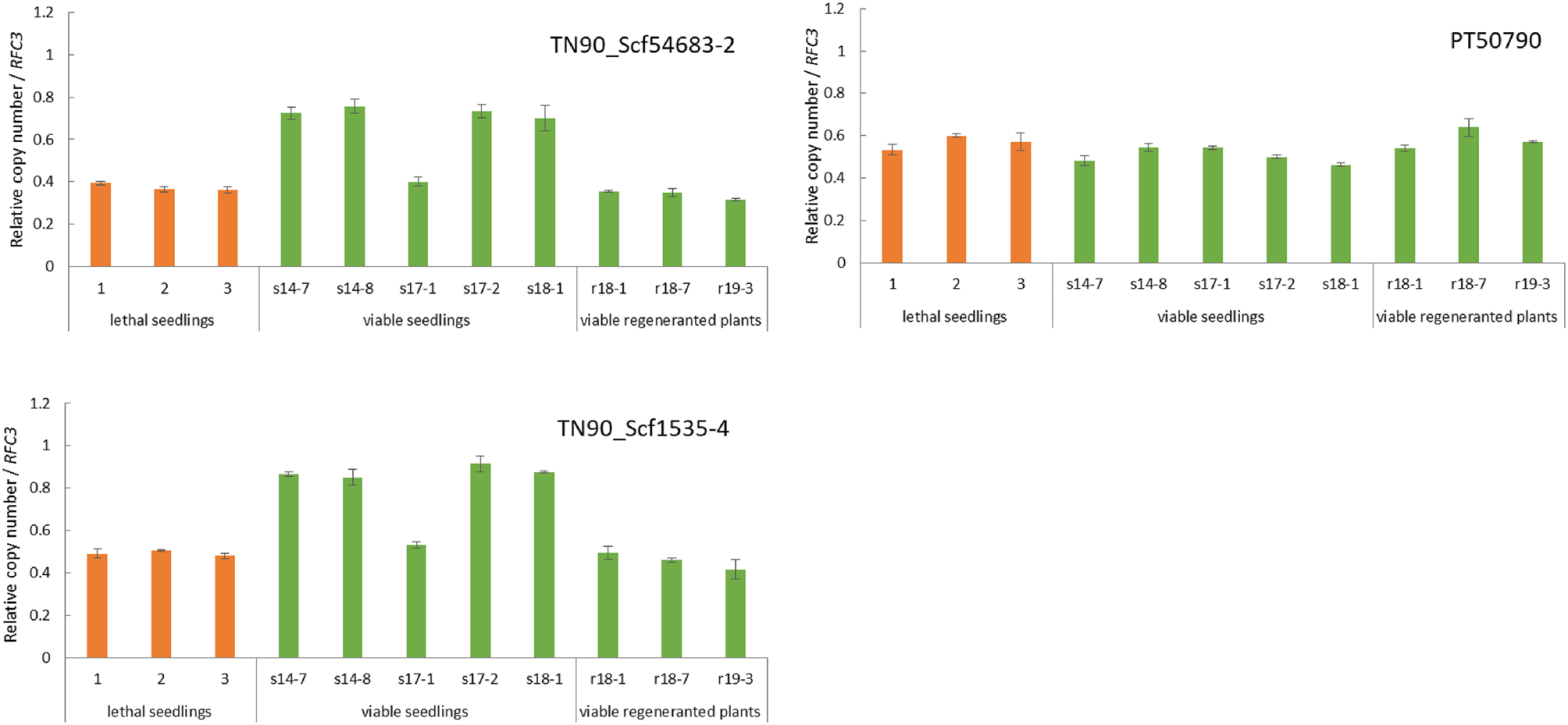
Mean (± SE) of relative copy number of each marker on the Q′ chromosome in lethal seedlings (No. 1–No. 3), viable seedlings lacking distal part of the Q chromosome (s14-7 ~ 8, s17-1 ~ 2, and s18-1), and viable regenerated plants lacking distal part of the Q chromosome (r18-1, r18-7, and r19-3). Quantitative Real-Time-Polymerase Chain Reaction (qRT-PCR) was used to determine copy number of each region on the Q′ chromosome relative to that of the *RFC3* gene. Data are shown as the mean ± SE from triplicate experiments for each DNA sample.

For amplification of the homoeologous chromosome of the Q chromosome (the Q′ chromosome) belonging to the T genome of *N. tabacum*, three sets of primers capable of specifically amplifying the SSRs sequences at both ends of the chromosome were selected (Fig. 5). Total DNA was extracted from 5 hybrid seedlings that overcame lethality and from 3 regenerated hybrid plants that overcame lethality, in which the SSRs of terminal DNA of the Q chromosome were not detected, and from 3 lethal hybrid seedlings. The copy number of the region amplified by each primer pair (normalized to a given amount of DNA) was then investigated by qPCR (Fig. 6). Specifically, a copy number was determined for the region amplified by two separate primer pairs (TN90_Scf1535-4 and TN90_Scf54683-2) designed based on the terminal DNA sequence of the Q′ chromosome, i.e., sequences homologous to the terminal DNA sequence of the Q chromosome, including the *Nt6549g30* locus. Notably, in 4 of the 5 hybrid seedlings that overcame lethality, the copy number of these selected regions was approximately twice that in lethal hybrid seedlings. The remaining hybrid seedling (s17-1) showed a value that was similar to that seen in the lethal hybrid seedling. On the other hand, the copy numbers of the PT50790-targeted domain (corresponding to the distal part of the Q′ chromosome, at the end opposite to the *Nt6549g30* locus) did not show a significant difference between lethal hybrid seedlings and hybrid seedlings that had overcame lethality. In addition, for the regenerated hybrid plants that overcame lethality, the copy number of each amplification region did not differ markedly from that of the lethal hybrid seedlings.

## Discussion

This study sought to elucidate the mechanism by which individual hybrid plants overcome hybrid lethality, whether spontaneously or when propagated artificially. To achieve this goal, we formulated and tested two (non-exclusive) working hypotheses based on previous findings regarding the lethality-overcoming phenomenon of products of a *N. suaveolens* × *N. tabacum* cross. Hypothesis 1 stated that seedlings that overcome lethality in the *N. suaveolens* × *N. tabacum* cross occur by formation of male gametes lacking the lethality-associated *Nt6549g30* gene, which is lost as a result of reciprocal translocations between homoeologous chromosomes during meiosis in *N. tabacum*. Hypothesis 2 tested whether culturing of *N. suaveolens* × *N. tabacum* seedlings in the presence of cytokinin yields regenerated plants that overcome hybrid lethality at high frequency; we proposed that hybrid lethality is overcome as a result of the loss of the distal part of the Q chromosome during culturing.

To examine Hypothesis 1, we first identified 16 surviving seedlings from 12,943 germinated *N. suaveolens* × *N. tabacum* seedlings. The results of RAPD-PCR and morphological observations revealed that some of the viable seedlings were indeed hybrids (i.e., possessing genomes from both parents) and showed extremely low expression levels of immune response-related genes, confirming these to be hybrid seedlings that had overcome lethality (Figs. 1, 2, 3, Tables 1, S1). Specifically, 11 hybrid seedlings that had overcome lethality were obtained, and the frequency of appearance of hybrid seedlings that overcame lethality in this cross combination was calculated to be 8.5 × 10^−4^. This value was close to 1.1 × 10^−3^, which is the frequency of appearance of hybrid seedlings that overcame lethality in a previous study of the *N. tabacum* × *N. africana* cross^20^.

**Figure 7:**
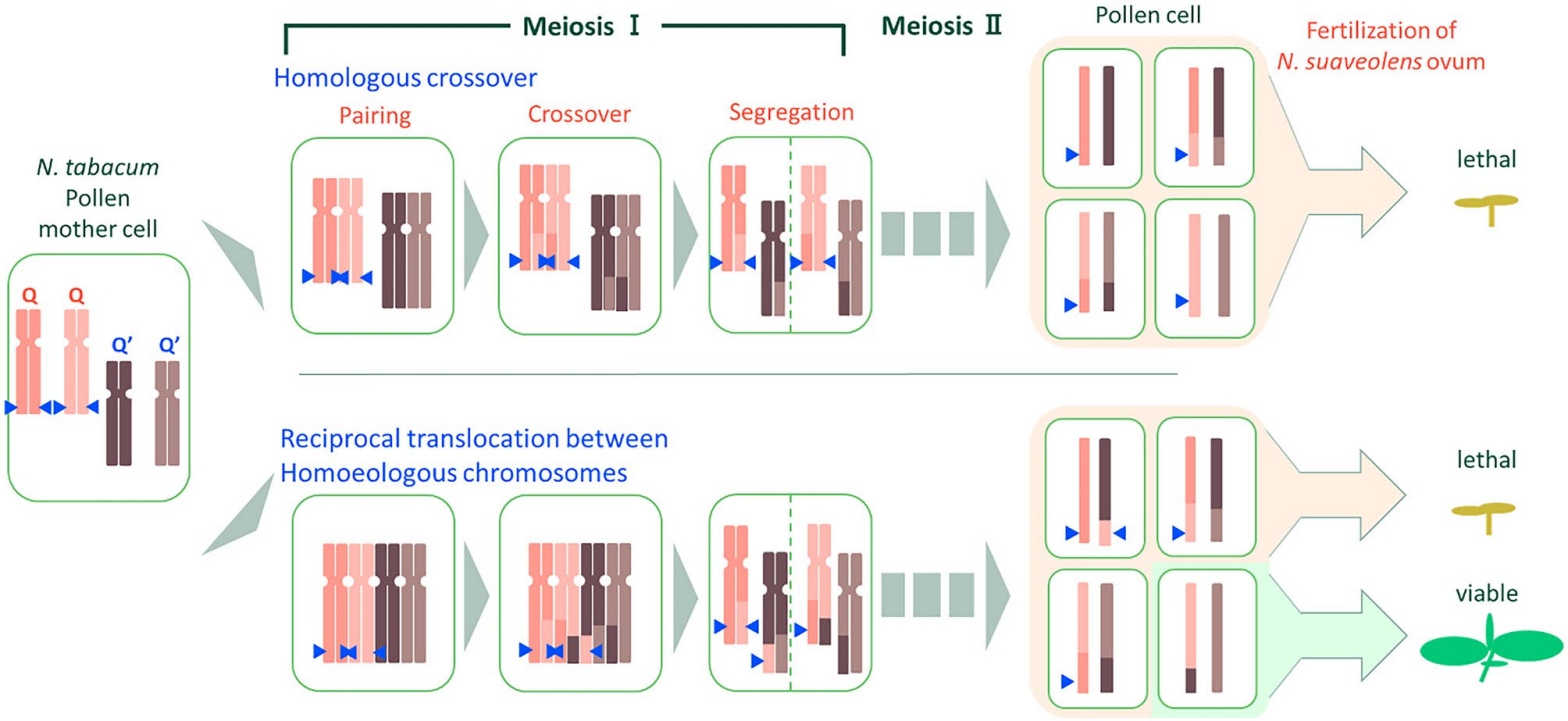
A proposed model for overcoming hybrid lethality by reciprocal translocation between homoeologous chromosomes in hybrid seedlings from the *N. suaveolens* × *N. tabacum* cross. Blue arrowhead indicates hybrid lethality-associated gene of *N. tabacum*.

PCR results showed that 5 of 8 of the seedlings that overcame lethality lacked the distal part of the Q chromosome, a region that contains *Nt6549g30* (Table 1). When qPCR was performed using two primer pairs (TN90_Scf1535-4 and TN90_Scf54683-2) designed to amplify sequences at the end of the Q′ chromosome that are homoeologous to the Q chromosome region containing *Nt6549g30* (Fig. 5), the copy number of the amplified region from 4 of the 5 individuals that overcame lethality was approximately twice that of seedlings that exhibited lethality (Fig. 6). Meanwhile, there was no difference in copy number of the DNA amplified by the PT50790 primer pair in the lethal seedlings and seedlings that overcame lethality. The PT50790 primer set is designed to amplify the end of the Q′ chromosome that is opposite to the end that the TN90_Scf1535-4 and TN90_Scf54683-2 primers (Fig. 5). *Nt6549g30* is not contained in the region of the Q chromosome that is homoeologous to this site on the Q′ chromosome. Therefore, in these 4 individuals that overcame lethality, it appears that the end of the Q chromosome that contains *Nt6549g30* was replaced due to a reciprocal translocation between the Q chromosome and its homoeologous chromosome (Q′). These data support the existence of a mechanism for overcoming lethality that is consistent with our Hypothesis 1. Figure 7 shows a suggested model, based on our results, for such a mechanism in the *N. suaveolens* × *N. tabacum* cross. In this model, a hybrid seedling that overcomes lethality is produced by fertilization of a female gamete from *N. suaveolens* by a male gamete from *N. tabacum*; notably, the male gamete has lost *Nt6549g30* due to reciprocal translocation between the distal regions of the Q and Q′ chromosomes during meiosis. This model is consistent with the manner in which lethality is overcome in *N. tabacum* × *N. africana*^20^ and in other interspecific crosses in the genus *Nicotiana* where *N. tabacum* is one of the parents.

GRAS-Di analysis of one hybrid (s17-2) that escaped lethality suggested that lethality was overcome by reciprocal translocation combined with qPCR result. Notably, an amplicon derived from the region extending to Q chromosomal DNA bp 81,360,673 was detected, but no amplicon derived from the DNA region beyond bp 81,497,158 was detected (Table S4). This result implies that the break-point of the reciprocal translocation detected in the hybrid seedling that overcame lethality (s17-2) is located within a region of about 136 kbp, which is located between bp 81,360,673 and bp 81,497,158 on the Q chromosome.

For one of the five seedlings that overcame lethality (s17-1), qPCR analysis showed that the copy number of the amplified region was the same as that of the lethal seedlings (Fig. 6). This observation implicated deletion of the distal part of the Q chromosome in overcoming lethality in this individual. Specifically, for s17-1, products amplified by the three primer pairs (PT52864, PT55075, and PT60178) that would span the SSR marker located near the center of the Q chromosome (at 62.25 cM) were not detected (Table 1). Therefore, reciprocal translocations between homoeologous chromosomes may be more likely to occur in the region near the end of the Q chromosome, while deletions may be more likely to occur in the region closer to the center of the chromosome. In synthetic allopolyploid *Brassica* progeny plants, both chromosomal deletions and reciprocal translocations (between homoeologous chromosomes) have been reported^31,32^. We postulate that the deletion observed at the distal part of the Q chromosome in this study may be the result of aberrant segregation and cleavage of chromosomes following synapsis between homoeologous chromosomes during meiosis in *N. tabacum*, which is allopolyploid.

In 3 individuals that overcame lethality (s14-1, s14-2, and s14-4), i.e., approximately 38% of the 8 seedlings that overcame lethality and were subjected to PCR, amplicons were detected for all of the primer pairs (Table 1). Therefore, it appeared that these individuals did not lack the lethality-associated gene on the *N. tabacum* side (*Nt6549g30*) as a result of reciprocal translocation or deletion of the distal part of the Q chromosome. In *N. tabacum* × *N. africana*, it has been suggested that about 37% of seedlings that overcome lethality have intact Q chromosomes^20^. Both *N. suaveolens* and *N. africana* are included in the Suaveolentes section and are thought to be derived from amphidiploid progenitors^33^. Based on these results, we hypothesized that reciprocal translocation or deletion of the chromosome segment on which the lethal gene is located can also occur in *N. suaveolens* and *N. africana*. Overcoming lethality in seedlings produced by *N. suaveolens* × *N. tabacum* and *N. tabacum* × *N. africana* crosses, in which no reciprocal translocation or deletion of the distal part of the Q chromosome was detected, may result from chromosomal mutations due to synapsis of homoeologous chromosomes. However, since the lethal genes in *N. suaveolens* and *N. africana* are unknown and the genomic sequences of these species have not yet been unpublished, this hypothesis cannot be tested at present. In addition, epigenetic suppression of expression or sequence destruction by transposon translocation in the genes responsible for hybrid lethality may also play a role.

To test Hypothesis 2, seedlings of *N. suaveolens* × *N. tabacum* were cultured in a medium containing a high concentration of cytokinin in order to obtain viable regenerated plants. The results of RAPD-PCR analysis and morphological observations showed that the resulting regenerated plants had genomes from both parents and confirm that these individuals were indeed hybrids. These plants also showed marked decreases in the expression levels of immune response-related genes. Therefore, we judged these individuals to be regenerated hybrid plants that had overcome lethality (Figs. 2, 3, 4, Tables 1, S1). In 3 (r18-1, r18-7, and r19-3) of the 12 regenerated plants that overcame lethality, PCR analysis showed that the distal part of the Q chromosome (the region containing *Nt6549g30*) had been lost, as seen in some of the seedlings that overcame lethality (Table 1). Additionally, qPCR analysis of these 3 individuals showed that the copy number of the amplified region was comparable to that of lethal seedlings (Fig. 6). These results suggested that the deletion of the distal part of the Q chromosome in these three individuals contributed to them overcoming lethality, an inference that is consistent with our Hypothesis 2. Hypothesis 2 was based on a previous study in which it was shown that ROS are generated in culture^28^ and that they are involved in chromosomal cleavage^29^. In the future, it will be necessary to verify that ROS are involved in the deletion of the distal part of the Q chromosome. Such confirmatory experiments will require demonstrating ROS production in hybrid seedlings that are cultured in a medium containing a high concentration of cytokinin, and then showing that inhibition of ROS production prevents the emergence of viable shoots.

In 9 of the 12 regenerated plants that overcame lethality, PCR analysis showed that amplicons were detected for all primer pairs (Table 1). Thus, in these individuals, it appears that lethality was overcome by a mechanism other than deletion of the distal part of the Q chromosome. In addition to recombination-associated events that result in chromosomal lesions, mutation by DNA methylation and by DNA base substitution and deletion is known to occur in plant tissue culture^28^. It also has been suggested that the supplementation of medium with high-concentration BAP induces mutation^34^. Furthermore, certain *R* genes have been reported to show frequent DNA base substitutions and deletions during mitosis^35^. It is therefore possible that these various genetic mutations occur in the genes associated with lethality in *N. suaveolens* and *N. tabacum*, resulting in the formation of viable shoots during tissue culture. It also is possible that ROS production under culture conditions induces deletion of the chromosomal region containing the genes associated with lethality in the *N. suaveolens* genome. To clarify these mutational mechanisms, it will be necessary to perform detailed sequence analysis of the *Nt6549g30* locus in regenerated plants that have overcome lethality, and to identify the chromosome and gene in the *N. suaveolens* genome that are associated with lethality.

In the present study, expression analysis of immune-response-related genes (e.g., *PAL1*, *PRB1-like*, *LOX1*, and *PDF-like protein 1*) was performed in the process of screening seedlings and regenerated plants that overcame lethality. In *A. thaliana*, the *PAL1* gene product is involved in the synthesis of salicylic acid^36^, and *PRB1* encodes a PR-1-like protein that is expressed in response to ethylene and methyl jasmonate exposure^35^. On the other hand, the *LOX1* gene product has been implicated in jasmonic acid synthesis^38^, and *PDF1.2* encodes a jasmonic acid-responsive defensin^38^. In the present work, the expression levels of these genes in seedlings and regenerated plants that overcame lethality were much lower than those in the lethal seedlings at 6 DAG (Fig. 3). These data strongly suggested that the seedlings and regenerated plants that overcome lethality lack the function of the gene associated with lethality that triggers the immune-response signal, a deficiency that presumably is due to some genetic variation.

Hybrid lethality is a type of reproductive isolation and a mechanism employed for maintaining species independence. However, the results of the present study suggest that chromosomal instability permits hybrids to overcome hybrid lethality, thereby escaping reproductive isolation. We postulate that this phenomenon may contribute to the development of new species in the process of plant evolution. In addition, hybrid lethality has been reported in a wide range of crop species^10^ and is a serious obstacle in hybrid breeding of crops. The findings obtained in this study may lead to a technique for artificially breaking reproductive isolation by inducing chromosomal instability.

In summary, we found that the loss of the distal part of the Q chromosome is involved in overcoming hybrid lethality in seedlings and in regenerated plants obtained by culturing hybrid seedlings produced from interspecific *Nicotiana* crosses. We propose that the loss of this factor is the result of reciprocal translocation and deletion between the Q chromosome and its homoeologous partner chromosome (Q′) in hybrid seedlings, and of a distal deletion in the Q chromosome in regenerated plants. These findings, which relate to the mechanism of breaking reproductive isolation, are likely to be important in the evolution of polyploid plant species and in the expansion of genetic resources available for cross-breeding.

## Materials and methods

### Sowing hybrid seeds and obtaining viable seedlings

Hybrid seeds of *N. suaveolens* × *N. tabacum* were obtained according to the method of Yamada et al.^39^. The seeds were surface-sterilized by immersion in 70% ethanol for 30 s, followed by immersion in 5% sodium hypochlorite for 20 min. After washing three times with sterile water on a clean bench, seeds were sown in half-concentration MS medium^40^ (0.5 × MS) that had been adjusted to pH 5.8 and supplemented with 1% sucrose and a gelling agent (0.2% Gelrite (FUJIFILM Wako) or 0.3% Phytagel (Sigma-Aldrich)). The medium containing the seeds then was incubated at 28 °C in an incubator with a photoperiod of 24 h. The number of seedlings that remained viable 20 days after germination was determined.

### Culturing hybrid seedlings in medium containing a high concentration of cytokinin, and obtaining viable shoots

After surface-sterilization as above, hybrid seeds of *N. suaveolens* × *N. tabacum* were sown in 0.5 × MS that had been adjusted to pH 5.8 and supplemented with 2.0 mg/L 6-benzylaminopurine (BAP) (Sigma-Aldrich), 1% sucrose, and a gelling agent (0.2% Gelrite or 0.3% Phytagel). The medium containing the seeds then was incubated at 28 °C in an incubator with a photoperiod of 24 h. Viable shoots that differentiated from the stem base after incubation for more than 1 month from sowing were excised, transplanted to 0.5 × MS without plant hormones, and rooted.

### DNA extraction

Leaves or stems were collected from hybrid parents, lethal seedlings, viable seedlings, and viable regenerated plants. Total DNA was extracted from these tissues using the cetyltrimethylammonium bromide (CTAB) method according to Yamada et al.^39^, or using a DNeasy Plant Mini Kit (QIAGEN) according to the manufacturer’s protocol. Purity and concentration of DNA samples were determined using NanoDrop Lite (Thermo Fisher Scientific) and Qubit 2.0 Fluorometer (Thermo Fisher Scientific) instruments.

### Confirmation of hybridity by RAPD-PCR

Hybridity was tested by RAPD-PCR with reference to Marubashi and Onosato^16^ and Tezuka et al*.*^41^. Six PCR primers were used, including OPA-1, OPA-5, OPA-9, OPA-11, OPA-12, and OPA-15 (Operon Technologies). KAPA Taq Extra Hotstart Ready mix with dye (Kapa Biosystems) was used for PCR. The PCR reaction solution was prepared by combining 10 μL of 2 × KAPA Taq EXtra HotStart ReadyMix with dye, 2 μL of OPA primer at 10 μM, and 20 ng of DNA; the solution then was adjusted to 20 μL with PCR-grade water and mixed. The PCR reaction solution was subjected to initial denaturation at 95 °C for 15 min, followed by 45 cycles of denaturation at 94 °C for 1 min, annealing at 35 °C for 2 min, and extension at 72 °C for 3 min, using an Applied Biosystems 2720 Thermal Cycler (Life Technologies). The PCR products were electrophoresed (along with a commercial DNA size marker) on a 2% or 3% agarose gel supplemented with 0.01% GelRed (Biotium) using TAE buffer (4.84 g/L tris (hydroxymethyl) aminomethane, 1.14 mL/L acetate, and 370 mg/L EDTA) as the electrophoresis buffer, and the gel was photographed under UV light to detect the PCR products.

### Confirmation of hybridity by morphological observation

Viable seedlings and regenerated plants were transplanted into pots containing Super Soil Mix A (Sakata Seed), acclimated in a constant temperature room at 25 °C for 16 h, and allowed to flower. Leaf and flower morphology and plant posture were compared with those of the parent strains.

### Expression analysis

Total RNA was extracted from cotyledons of lethal seedlings, true leaves of viable seedlings, and regenerated *N. suaveolens* × *N. tabacum* plants using RNAiso Plus (Takara Bio) according to the method of Shinozaki et al*.*^42^. Lethal seedlings were cultivated according to the method described above for “Sowing hybrid seeds and obtaining viable seedlings”; the sample consisted of the cotyledons collected from 30 to 50 individuals at the 6-DAG stage. Seedlings and regenerated plants that overcame lethality were cultivated according to the method described above for “Confirmation of hybridity by morphological observation”, and young true leaves were sampled. Purity and concentration of RNA samples were determined using Nanodrop and Qubit instruments. A PrimerScript RT reagent Kit with gDNA Eraser (Takara Bio) was used for synthesis of cDNA, and the initial template cDNA was adjusted to 10 ng/μL. The immune-response genes for *N. tabacum*, which have high homology with those of *A. thaliana*, were used as the targets for expression analysis (Table S5). Primer pairs targeting each gene were designed using Primer3Plus (https://primer3plus.com/) and NetPrimer (PREMIER Biosoft; http://www.premierbiosoft.com/netprimer/) software programs (Table S5). For real-time quantitative reverse transcription (qRT) -PCR, a mixture corresponding to 5 µL of KAPA SYBR FAST Universal 2 × qPCR Master Mix (Kapa Biosystems), 0.2 µL of 10 µM Forward primer, 0.2 µL of 10 µM Reverse primer, 3.6 µL of PCR-grade water, and 1 µL of cDNA solution was generated and distributed to each well of a 0.2-mL 48-well PCR plate. The PCR reaction solution was subjected to initial denaturation at 95 °C for 10 min followed by 40 cycles of denaturation at 94 °C for 10 s, and annealing and extension at 60 °C for 30 s using an Eco Real-Time PCR System (Illumina). Finally, melting curve analysis was performed from 60 °C to 95 °C and the plate was incubated at 4 °C. The mRNA copy number of each gene in 10 ng of total RNA of each sample was determined by comparison to a standard curve. The mRNA copy number of *RFC3*, a housekeeping gene, was determined as an internal standard and used for normalization to determine relative transcript amounts. qRT-PCR analysis was performed in triplicate; the resulting data were used to calculate mean transcript levels.

### Detection of *Nt6549g30* and SSR markers on the Q chromosome

The nucleotide sequence of *Nt6549g30* was obtained from Ma^12^, and gene-specific primers *Nt6549g30*-1 and *Nt6549g30*-3 were designed using NetPrimer software (Fig. 5, Table S5). The SSR markers on the Q chromosome (Linkage group No. 11) were obtained from Bindler et al*.* ^43,44^, and corresponded to primer pairs that amplify products from *N. tabacum*, but not from *N. suaveolens*, or those that generate amplicons of distinct sizes in the two species (Fig. 1, Table S5). These PCR amplifications used the KAPA Taq EXtra PCR Kit (Kapa Biosystems), with each 10-µL reaction comprising 2.0 μL of 5 × KAPA Taq EXtra Buffer, 0.6 μL of 25 mM MgCl_2_, 0.2 μL of 10 mM dNTP Mix, 0.5 μL of 10 μM Forward primer, 0.5 μL of 10 μM Reverse primer, 0.5 μL of 5 U/μL KAPA Taq EXtra DNA Polymerase, 20 ng of DNA, and PCR-grade water to volume. Alternatively, the reactions used the KAPA Taq EXtra HotStart ReadyMix with dye, with each 10-µL reaction comprising 5 μL of 2 × KAPA Taq EXtra HotStart ReadyMix with dye, 0.5 μL of 10 μM Forward primer, 0.5 μL of 10 μM Reverse primer, 20 ng of DNA, and PCR-grade water to volume. For detection of *Nt6549g30*, the PCR reaction solutions were subjected to initial denaturation at 94 °C for 5 min, followed by 30 cycles of denaturation at 94 °C for 0.5 min, annealing at 55 °C for 0.5 min, and extension at 72 °C for 1 min. For detection of SSR markers, the PCR reaction solutions were subjected to initial denaturation at 94 °C for 3 min followed by 35 cycles of denaturation at 94 °C for 0.5 min, annealing at 55 °C for 0.5 min, and extension at 72 °C for 1 min. The PCR products were electrophoresed and visualized as described above for RAPD-PCR products.

### GRAS-Di

GRAS-Di analysis was performed under contract at GeneBay, Inc. Among the obtained GRAS-Di amplicons, those specific to *N. tabacum* reference genomic sequence according to Edwards et al.^45^ (ftp://ftp.solgenomics.net/genomes/Nicotiana_tabacum/edwards_et_al_2017/assembly/Nitab-v4.5_genome_Chr_Edwards2017.fasta), those specific to the scaffolds included in the Q chromosome (Linkage group No. 11), and those with no mismatch were selected. For each individual, the marker with the number of sequenced reads judged to be 0 was defined as “no detection”, and the marker for which the number of sequenced reads was judged to be greater than 0 was defined as “detection”.

### Analysis of the copy number of the SSR marker on the Q homoeologous chromosome

The SSR marker on the homoeologous chromosome of the Q chromosome (Linkage group No. 13, Edwards et al.^45^) from Bindler et al*.* ^44^ that failed to exhibit PCR amplification in *N. suaveolens* was selected and used for qPCR (Fig. 5, Table S5). In addition, Primer-BLAST (https://www.ncbi.nlm.nih.gov/tools/primer-blast/) was used to identify the scaffold (Sierro et al.^46^) that mapped to the homoeologous partner of the Q chromosome and contained the SSR marker. Flanking sequences from this scaffold were used to design primers (thereby defining DNA markers) that generated products from *N. tabacum*, but not from *N. suaveolens* (Fig. 5, Table S5). Real-time q-PCR was performed according to the method described above in “Expression analysis” using (as templates) the total DNA of lethal seedlings, viable seedlings, and regenerated plants in which no SSR marker corresponding to the distal part of the Q chromosome was detected. These measurements were repeated three times and the resulting data were used to calculate mean values.

## Supplementary Information

Supplementary Information

## Data Availability

All data generated or analyzed during this study are included in this published article and its Supplementary Information files.
