# Publisher Correction: Analysis of the possible cytogenetic mechanism for overcoming hybrid lethality in an interspecific cross between *Nicotiana suaveolens* and *Nicotiana tabacum*

**DOI:** 10.1038/s41598-021-95580-9

**Published:** 2021-08-05

**Authors:** Kouki Nakata, Hiroki Nagashima, Natsuki Inaba, Haruka Yamashita, Yoshihito Shinozaki, Motoki Kanekatsu, Wataru Marubashi, Tetsuya Yamada

**Affiliations:** 1 Graduate School of Agricultural Science, Tokyo University of Agriculture and Technology, Tokyo, 183-0054 Japan; 2 Faculty of Agricultural Science, Meiji University, Kanagawa, Japan; 3 Present Address: Division of Evolutionary Genetics, National Institute of Genetics, Shizuoka, Japan; 4 Present Address: Department of Genetics, The Graduate University for Advanced Studies (SOKENDAI), Shizuoka, Japan

Correction to: *Scientific Reports* 10.1038/s41598-021-87242-7, Published online 09 April 2021

The original version of this Article contained errors in Table 1, where the placement of sample names was incorrect for “Lethal seedlings” and “Viable seedlings”. The correct and incorrect values appear below.

Incorrect:

**Table:** 

Type of primer pair	Name of primer pair	Distance(cM)^c^	*N*. *suaveolens*	*N. tabacum*	Lethal seedlings			Viable seedlings							
					1		3	2	s14-2	s14-4	s14-7	s14-8	s17-1	s17-2	S18-1
SSR^a^	PT20383	38.6	−	+	+	+	+	+	+	+	+	+	+	+	+
	PT1348	49.0	−	+	+	+	+	+	+	+	+	+	+	+	+
	PT54570	75.9	−	+	+	+	+	+	+	+	+	+	+	+	+
	PT52864	99.5	−	+	+	+	+	+	+	+	+	+	−	+	+
	PT55075	105.7	−	+	+	+	+	+	+	+	+	+	−	+	+
	PT60178	109.6	−	+	+	+	+	+	+	+	+	+	−	+	+
	PT53094	117.6	−	+	+	+	+	+	+	+	+	−	−	−	−
	PT30342	122.0	−	+	+	+	+	+	+	+	−	−	−	−	−
	PT30365	122.8	−	+	+	+	+	+	+	+	−	−	−	−	−
	PT52778	124.5	−	+	+	+	+	+	+	+	−	−	−	−	−
Gene	Nt6549g30-1^b^	N.A	−	+	+	+	+	+	+	+	−	−	−	−	−
	Nt6549g30-3^b^		−	+	+	+	+	+	+	+	−	−	−	−	−

Correct:

**Table 1:** Detection of products, amplified by SSR and *Nt6549g30* primer pairs, from the Q chromosome (Linkage group No. 11) of *N. tabacum* in lethal seedlings, viable seedlings, and viable regenerated plants of the *N. suaveolens* × *N. tabacum* cross.

Type of primer pair	Name of primer pair	Distance(cM)^c^	*N. suaveolens*	*N. tabacum*	Lethal seedlings			Viable seedlings							
					1	2	3	s14-1	s14-2	s14-4	s14-7	s14-8	s17-1	s17-2	s18-1
SSR^a^	PT20383	38.6	**−**	**+**	**+**	**+**	**+**	**+**	**+**	**+**	**+**	**+**	**+**	**+**	**+**
	PT1348	49.0	**−**	**+**	**+**	**+**	**+**	**+**	**+**	**+**	**+**	**+**	**+**	**+**	**+**
	PT54570	75.9	**−**	**+**	**+**	**+**	**+**	**+**	**+**	**+**	**+**	**+**	**+**	**+**	**+**
	PT52864	99.5	**−**	**+**	**+**	**+**	**+**	**+**	**+**	**+**	**+**	**+**	**−**	**+**	**+**
	PT55075	105.7	**−**	**+**	**+**	**+**	**+**	**+**	**+**	**+**	**+**	**+**	**−**	**+**	**+**
	PT60178	109.6	**−**	**+**	**+**	**+**	**+**	**+**	**+**	**+**	**+**	**+**	**−**	**+**	**+**
	PT53094	117.6	**−**	**+**	**+**	**+**	**+**	**+**	**+**	**+**	**+**	**−**	**−**	**−**	**−**
	PT30342	122.0	**−**	**+**	**+**	**+**	**+**	**+**	**+**	**+**	**−**	**−**	**−**	**−**	**−**
	PT30365	122.8	**−**	**+**	**+**	**+**	**+**	**+**	**+**	**+**	**−**	**−**	**−**	**−**	**−**
	PT52778	124.5	**−**	**+**	**+**	**+**	**+**	**+**	**+**	**+**	**−**	**−**	**−**	**−**	**−**
Gene	*Nt6549g30*-1^b^	N.A	**−**	**+**	**+**	**+**	**+**	**+**	**+**	**+**	**−**	**−**	**−**	**−**	**−**
	*Nt6549g30*-3^b^		**−**	**+**	**+**	**+**	**+**	**+**	**+**	**+**	**−**	**−**	**−**	**−**	**−**

‘ + ’ indicates the presence of allele of N. tabacum, ‘-’ indicates the absence of allele of N. tabacum. N.A., not applicable.

^a^ Bindler et al. ^43^,^44^.

^b^ Closely linked to PT30342, as confirmed by linkage analysis (Ma ^12^).

^c^ Genetic distance from the marker amplified by primer pair PT54560.

