# Supplementary material for: Analysis of the possible cytogenetic mechanism for overcoming hybrid lethality in an interspecific cross between Nicotiana suaveolens and Nicotiana tabacum
Source: Sci Rep. 2021 Apr 9;11:7812. doi: 10.1038/s41598-021-87242-7 (PMC8035154; doi:10.1038/s41598-021-87242-7)
Supplement: Supplementary file 1 — Supplementary Information [file 41598_2021_87242_MOESM1_ESM.docx]

**Supporting Information**

**Table S1.** Number of *N. suaveolens*- and *N. tabacum*-specific bands from Randomly Amplified Polymorphic DNA-Polymerase Chain Reaction (RAPD-PCR). Products obtained from each viable seedling and viable regenerated plant were separated by agarose gel electrophoresis and enumerated by visual inspection.


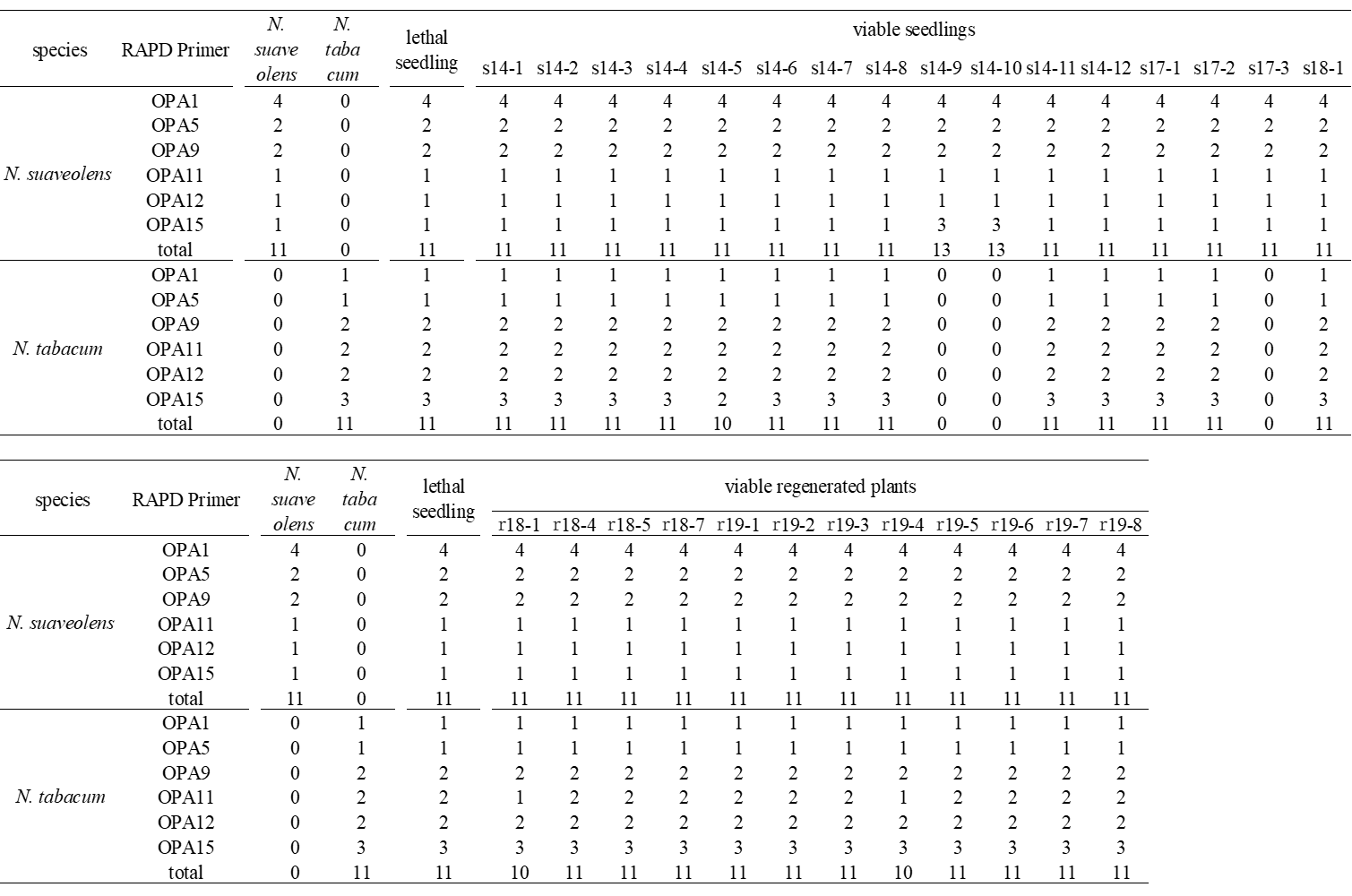


**Table S2.** Phenotype of each viable seedling and each viable regenerated plant. See also Figure 1 and Figure 4.


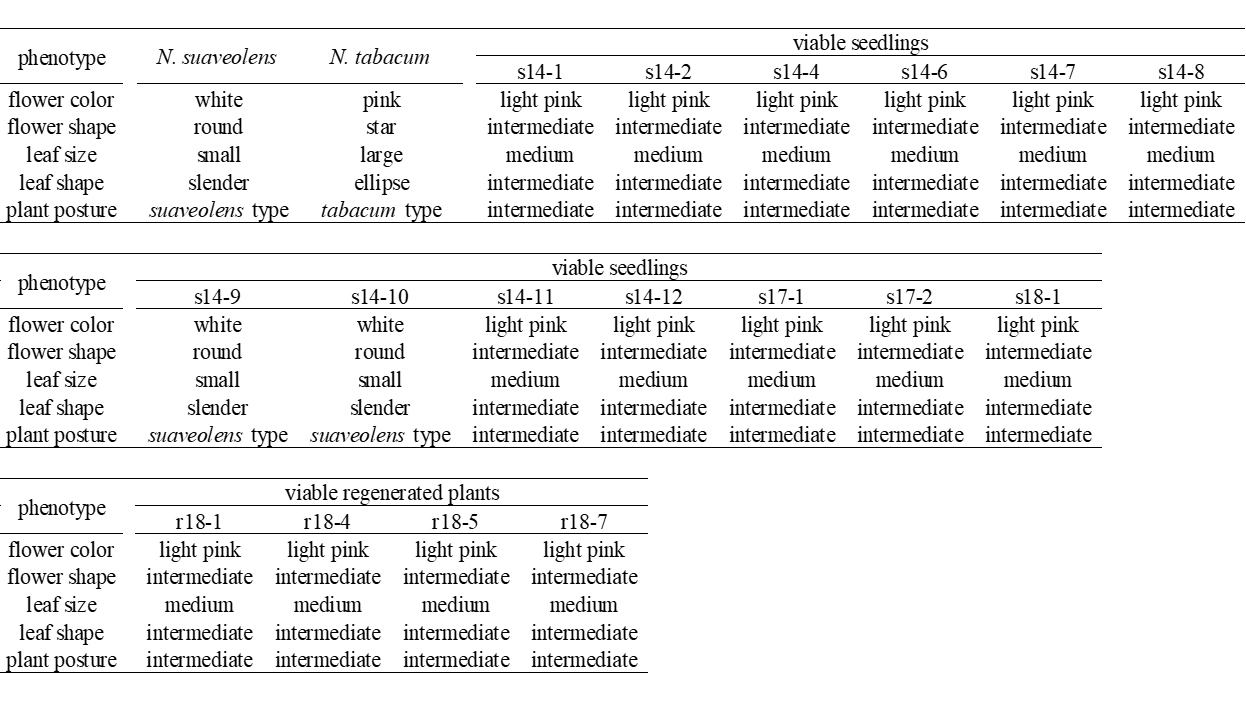


**Table S3.** Genes targeted for expression analysis.

a: Corresponding to National Center for Biotechnology Information (NCBIフォームの終わり) database (https://www.ncbi.nlm.nih.gov/)

b: Corresponding to The Arabidopsis Information Resource (TAIR) database (https://www.arabidopsis.org/index.jsp)

**Table S4.** GRAS-Di on the Q chromosome (Linkage group No. 11) in lethal seedlings and viable seedling No. s17-2 from the *N. suaveolens* × *N. tabacum* cross. ‘+’ indicates the presence of allele of *N. tabacum*, ‘-’ indicates the absence of allele of *N. tabacum.*

**Table S5.** Primers used for this study.

a: Bindler *et al*.^43, 44^
